# The Volatile Anesthetic Isoflurane Increases Endothelial Adenosine Generation via Microparticle Ecto-5′-Nucleotidase (CD73) Release

**DOI:** 10.1371/journal.pone.0099950

**Published:** 2014-06-19

**Authors:** Mihwa Kim, Ahrom Ham, Katelyn Yu-Mi Kim, Kevin M. Brown, H. Thomas Lee

**Affiliations:** Department of Anesthesiology, College of Physicians and Surgeons of Columbia University, New York, New York, United States of America; University of Cincinnati, United States of America

## Abstract

Endothelial dysfunction is common in acute and chronic organ injury. Isoflurane is a widely used halogenated volatile anesthetic during the perioperative period and protects against endothelial cell death and inflammation. In this study, we tested whether isoflurane induces endothelial ecto-5′-nucleotidase (CD73) and cytoprotective adenosine generation to protect against endothelial cell injury. Clinically relevant concentrations of isoflurane induced CD73 activity and increased adenosine generation in cultured human umbilical vein or mouse glomerular endothelial cells. Surprisingly, isoflurane-mediated induction of endothelial CD73 activity occurred within 1 hr and without synthesizing new CD73. We determined that isoflurane rapidly increased CD73 containing endothelial microparticles into the cell culture media. Indeed, microparticles isolated from isoflurane-treated endothelial cells had significantly higher CD73 activity as well as increased CD73 protein. *In vivo*, plasma from mice anesthetized with isoflurane had significantly higher endothelial cell-derived CD144+ CD73+ microparticles and had increased microparticle CD73 activity compared to plasma from pentobarbital-anesthetized mice. Supporting a critical role of CD73 in isoflurane-mediated endothelial protection, a selective CD73 inhibitor (APCP) prevented isoflurane-induced protection against human endothelial cell inflammation and apoptosis. In addition, isoflurane activated endothelial cells Rho kinase evidenced by myosin phosphatase target subunit-1 and myosin light chain phosphorylation. Furthermore, isoflurane-induced release of CD73 containing microparticles was significantly attenuated by a selective Rho kinase inhibitor (Y27632). Taken together, we conclude that the volatile anesthetic isoflurane causes Rho kinase-mediated release of endothelial microparticles containing preformed CD73 and increase adenosine generation to protect against endothelial apoptosis and inflammation.

## Introduction

More than 1 trillion endothelial cells in human body cover the entire circulatory system and play an integral role in maintaining homeostasis of all organs [1]. Endothelial cells regulate vascular tone, angiogenesis, coagulation and release several critically important autocrine and paracrine compounds including sphingosine 1-phosphate, adenosine, nitric oxide, and prostaglandins [2], [3]. Furthermore, endothelial cells release microparticles originating from plasma membrane and these microparticles contain many biological active compounds including cell surface enzymes, membrane receptors as well as lipoproteins, RNAs and microRNAs [4]. Clinically, endothelial dysfunction contributes to acute and chronic vascular and organ injury [2], [3]. Indeed, endothelial cell death occurs frequently after ischemia and reperfusion injury, diabetes, coronary artery disease and sepsis [3], [5], [6].

Volatile anesthetic is administered to virtually all patients subjected to general anesthesia making it one of the most frequently used medications during the perioperative period [7]. Volatile anesthetics have non-anesthetic effects on the heart, vasculature and respiratory system by regulating blood pressure, heart rate, airway tone and systemic vascular resistance [8], [9]. In addition, volatile anesthetics have anti-necrotic and anti-inflammatory effects and protect against ischemia reperfusion injury of the heart, kidney and intestine [9]–[11]. Furthermore, volatile anesthetics attenuate the hyperactive systemic inflammatory response during sepsis [12].

After inhalation, volatile anesthetics are first taken up by the pulmonary circulatory system and all endothelial cells in the body are rapidly exposed. We and others previously demonstrated that volatile anesthetics protect against endothelial cell necrosis, apoptosis and inflammation due to hypoxia, lipopolysaccharide and cytokine exposure [13]–[17]. However, the detailed signaling mechanisms of volatile anesthetic-mediated endothelial protection remain incompletely understood. We recently demonstrated that one of the most widely used volatile anesthetic isoflurane (2-chloro-2-(difluoromethoxy)-1,1,1-trifluoro-ethane) induces the expression of ecto-5′-nucleotidase (CD73) in renal proximal tubular cells [18]. Isoflurane-mediated induction of CD73 activity with subsequently increased synthesis of adenosine reduces proximal tubular necrosis, apoptosis and inflammation after renal ischemia and reperfusion injury. In this study, we tested whether isoflurane stimulates endothelial CD73 activity and generates adenosine to protect against endothelial cell death and inflammation. To our surprise, isoflurane did not induce new CD73 synthesis in endothelial cells as observed in renal proximal tubular cells. Instead, we determined that isoflurane activates endothelial cell Rho kinase to release preformed CD73 contained within endothelial microparticles to increase adenosine generation to protect endothelial apoptosis and inflammation.

## Methods and Materials

### Ethics Statement

All animal work was approved by Columbia University Institutional Animal Care and Use Committee.

### Endothelial Cell Culture and Exposure to Isoflurane

Immortalized human umbilical vein endothelial cells (EA.hy926, American Type Culture Collection, Manassas, VA) cells were grown in high-glucose DMEM plus 10% FBS. Immortalized mouse glomerular endothelial cells (GENC) were obtained from Dr. M. Madaio (Georgia Regents University) and grown in low glucose DMEM/Ham’s F12 medium plus 10% FBS, 2 mM l-glutamine, and 10 mM HEPES [19]. Cells were plated in 6-well plates when 80% confluent and used in the experiments described below when confluent after 24 hr serum deprivation.

Endothelial cells were exposed to isoflurane (Abbott Laboratories, Chicago, IL) as described previously [13]. In brief, endothelial cells were placed in an air-tight, 37°C, humidified modular incubator chamber (Billups-Rothenberg, Del Mar, CA) and exposed to 0–2.5% (0–2 MAC) isoflurane (where 1 MAC is defined as the percent concentration in the alveolus of an inhaled anesthetic agent required to prevent 50% of subjects from moving in response to a painful stimulus when used as the sole anesthetic) mixed with 95% air+5% CO2 (carrier gas). Exposure to isoflurane lasted between 0 to 16 hr. To inhibit CD73 or Rho kinase activity, some endothelial cells were pretreated with 100 µM α,β-methylene ADP (APCP, a selective CD73 inhibitor, Sigma, St Louis, MO) or with 10 µM Y27632 (a selective Rho Kinase inhibitor, Sigma, St Louis, MO) 30 min. before isoflurane treatment [20].

### Microparticle Isolation and Flow Cytometry

After Columbia University Institutional Animal Care and Use Committee approval, we anesthetized adult male C57BL/6 (Harlan, Indianapolis, IN) to 4 hr of equipotent doses of either pentobarbital or isoflurane (1.2% or ∼1 MAC) as described previously [18]. The mice were placed on a heating pad under a warming light to maintain body temperature ∼36–38°C. After 4 hr, animals were killed and plasma isolated for microparticle isolation.

Microparticles were isolated by differential centrifugation as described by Amabile *et al*. [21]. Endothelial cell culture medium or mouse plasma was centrifuged once at 1,000×g for 10 min to remove the cell pellet and the resulting supernatant was centrifuged at 20,000×g for 40 min. The resulting pellet containing microparticles was analyzed for CD73 activity as well as CD73 immunoblotting.

We also subjected isolated endothelial and mouse plasma microparticles to flow cytometric analysis with a Quanta SC flow cytometer (Beckman Coulter, Miami, FL). To determine the relative expression of CD73, microparticles isolated from endothelial cell culture media were stained with Annexin V (Beckman Coulter, Miami, FL) and CD73 (BD Biosciences, San Jose, CA) for 5 min at room temperature. Mouse plasma microparticles were stained with CD73 and CD144 (an endothelial cell marker, BD Biosciences, San Jose, CA) to determine the CD73 expression in endothelial microparticles.

### Induction of Endothelial Cell Apoptosis and Inflammation

After exposure to 2.5% isoflurane or with carrier gas for 1 hr, EA.hy9262 cells were exposed to tumor necrosis factor-alpha (TNF-α, 20 ng/ml) plus cycloheximide (10 µg/ml) for 16 hr to induce apoptosis or to TNF-α (20 ng/ml) for 6 hr to induce inflammation as described previously [18]. Apoptosis was assessed by detecting poly-(adensosine diphosphate-ribose)-polymerase (PARP) and caspase 3 fragmentations as described [22], [23]. Inflammation was assessed by measuring mRNAs encoding markers of inflammation including TNF-α, ICAM-1 and VCAM-1 (Table 1) with RT-PCR as described previously [18], [24].

**Table 1:** Primers used to amplify cDNAs based on published GenBank sequences for human.

Primers	Sequence (Sense/Antisense)	Product Size (bp)	Cycle Number	Annealing Temp (°C)
TNF-α	5′-CGGGACGTGGAGCTGGCCGAGGAG-3′	355	24	68
	5′-CACCAGCTGGTTATCTCTCAGCTC-3′			
ICAM-1	5′-GCAGACAGTGACCATCTACAGC-3′	400	16	60
	5′-GCCATCCTTTAGACACTTGAGC-3′			
VCAM-1	5′-TCTTGTTTGCCGAGCTAAATTA-3′	364	22	55
	5′-TAAATGGTTTCTCTTGAACAA-3′			
CD73	5′-CCA ATT CTG AGT GCA AAC AT-3′	315	23	62
	5′-CCT CCC ACC ACG ACG TCC AC-3′			
GAPDH	5′-ACCACAGTCCATGCCATCAC-3′	450	15	65
	5′-CACCACCCTGTTGCTGTAGCC-3′			

bp, base pairs; GAPDH, glyceraldehyde 3-phosphate dehydrogenase; ICAM-1, intercellular adhesion molecule-1; TNF-α, tumor necrosis factor-alpha; VCAM-1, vascular cell adhesion molecule-1; CD73, Ecto-5′-nucleotidase. Respective anticipated PCR product size (bp, base pairs), PCR cycle number for linear amplification, and annealing temperatures used for each primer are also provided.

### Detection of Endothelial CD73 mRNA and Protein Expression

We measured mRNA encoding human CD73 after isoflurane treatment as described [25] with primers listed in Table 1. In addition, EA.hy9262 cell lysates were collected for immunoblotting analyses of CD73 (Santa Cruz Biotechnologies, Santa Cruz, CA) and β-actin (internal protein loading control, Sigma, St Louis, MO) as described previously after isoflurane treatment [25].

### HPLC to Measure Endothelial Cell Adenosine Generation

EA.hy9262 or mouse glomerular endothelial cell culture media were collected after isoflurane treatment and assayed for adenosine by HPLC as described [18]. Adenosine was quantified on a C18 reversed-phase column with a binary low-pressure gradient elution system with a UV detector set to 254 nm as described [26]. Adenosine deaminase activity and adenosine uptake were inhibited with 10 µM erythro-9-(2-hydroxy-3-nonly)adenine (EHNA) and 10 µM dipyridamole, respectively.

### Endothelial Cell Lysate or Microparticle CD73 Activity Assay

CD73 activity was measured by tracking the conversion of AMP to adenosine with or without 100 µM APCP using a modified protocol according to Gelain *et al*. [27].

### Rho Kinase Activity Assay and Phospho-myosin Light Chain Immunoblotting

After 30 min treatment with 2.5% isoflurane or with carrier gas, we measured EA.hy9262 cell Rho kinase activity with a commercial assay that measures myosin phosphatase target subunit-1 (MYPT-1) Threonine residue 696 phosphorylation (Millipore, Billerica, MA). We also assessed Rho kinase activation by detecting myosin light chain (MLC) Serine-19 phosphorylation. EA.hy9262 cell lysates were probed with anti-phosphor-MLC 2 antibody as well as total-MLC 2 antibody (1∶10,000 anti-MLC20 (:1000, Cell Signaling Technology, Danvers, MA) and subjected to immunoblotting as described [18].

### Statistical Analysis

The data were analyzed with Student’s t-test when comparing means between 2 groups or with one way analysis of variance plus TUKEY’s post hoc multiple comparison test to compare mean values across multiple treatment groups. All data are expressed throughout the text as means ± SEM.

## Results

### Isoflurane Increases Adenosine Generation in Cultured Endothelial Cells

**Figure 1:**
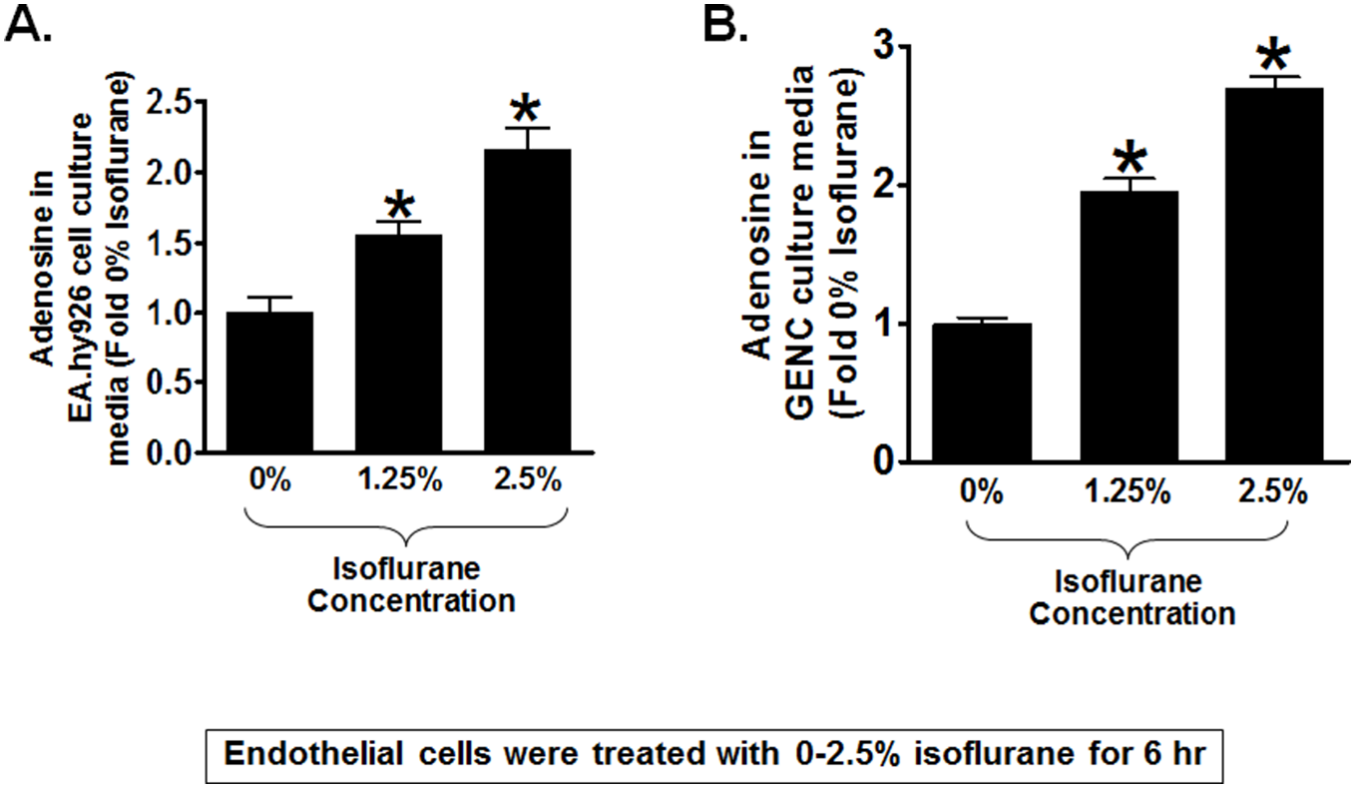
Isoflurane increases adenosine generation in cultured endothelial cells. Adenosine in media from human umbilical vein endothelial cells (EA.hy926, A) or mouse glomerular endothelial cells (GENC, B) measured with high pressure liquid chromatography. Isoflurane treatment (0–2.5%) for 6 hr increased adenosine concentrations when compared to carrier gas-treated controls (N = 5). Data are presented as means ± SEM. *P<0.05 vs. carrier gas-treated controls. Error bars represent 1 SEM.

We first determined whether isoflurane treatment increases adenosine generation in endothelial cells. Human umbilical vein (EA.hy9262) or mouse glomerular endothelial cells (GENC) were treated with 0–2.5% isoflurane for 6 hr and we measured adenosine levels in cell culture media with HPLC as described previously [18]. We determined that isoflurane treatment significantly increased adenosine levels in human and mouse endothelial cell culture media when compared with carrier gas-treated (room air plus 5% CO_2_) cells (Figure 1A).

### Isoflurane Transiently Increases CD73 Activity in Cultured Endothelial Cells

**Figure 2:**
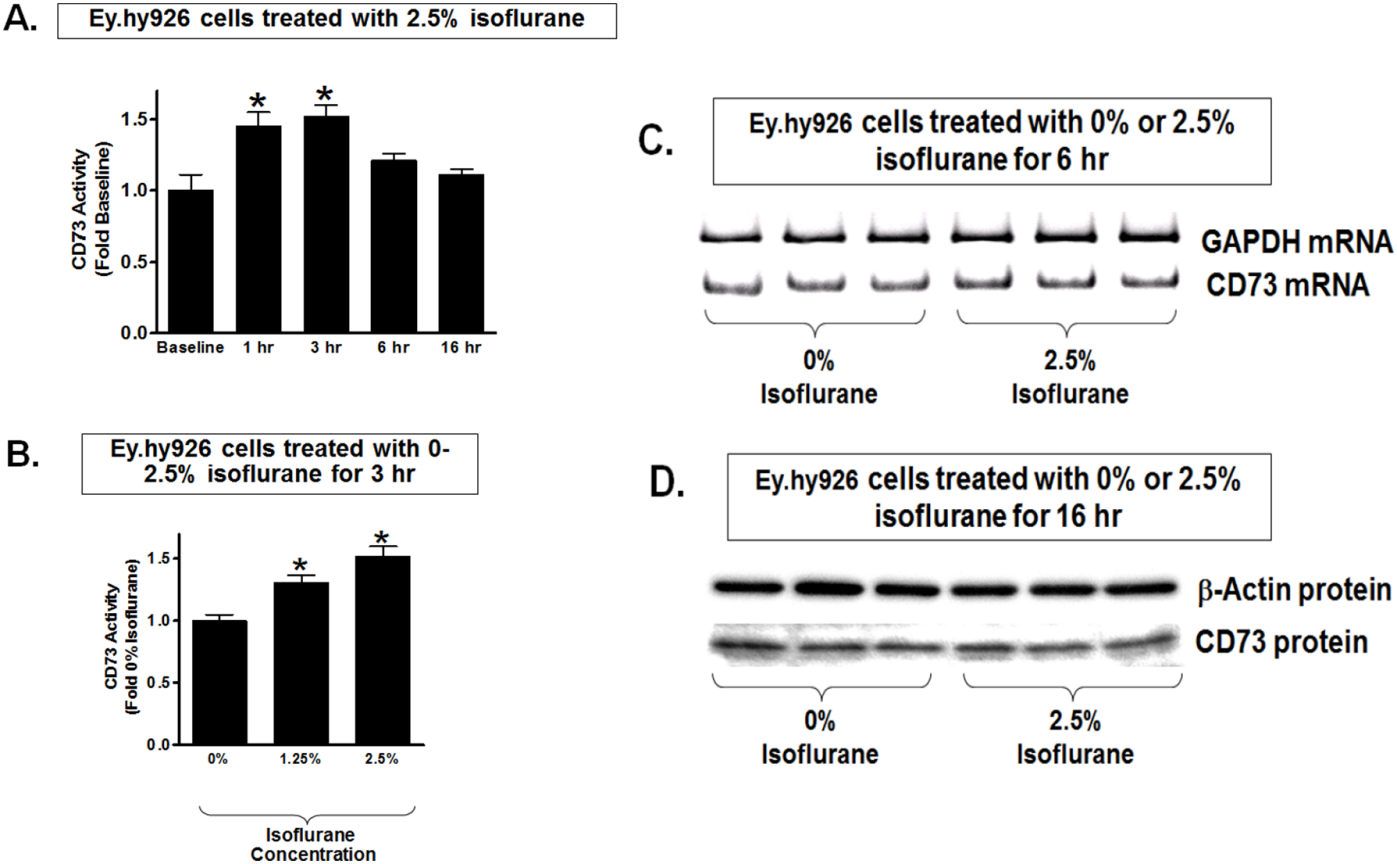
Isoflurane transiently increases CD73 activity without changing CD73 synthesis in cultured endothelial cells. A. Human umbilical vein endothelial (EA.hy926) cells treated with 2.5% isoflurane showed a significant but transient induction of CD73 activity. CD73 activity peaked at 3 hr and then decreased to near baseline at 6–16 hr after isoflurane treatment (N = 6–8). B. Isoflurane treatment for 3 hr caused dose-dependent increase in CD73 activity in EA.hy926 cells compared to carrier gas-treated cells (N = 4–5). Data are presented as means ± SEM. *P<0.05 vs. CD73 activity measured at baseline (A) or in cells treated with 0% isoflurane (B). C and D. Representative images for CD73 mRNA (RT-PCR) and protein (immunoblotting) expression in EA.hy926 cells. EA.hy926 cells were treated with carrier gas or with 2.5% isoflurane for 6 hr (C) or for 16 hr (D). Isoflurane treatment did not increase CD73 mRNA or protein expression in EA.hy926 cells. Representative of 3–4 experiments.

The next set of experiments determined whether isoflurane stimulates CD73 activity to increase adenosine generation in cultured endothelial cells. EA.hy9262 cells had significantly increased cell surface CD73 activity (conversion of AMP to adenosine) 1 to 3 hr after treatment with 2.5% isoflurane (Figure 2A). We also observed increased CD73 activity in EA.hy9262 cells with 1.25–2.5% isoflurane treatment for 3 hr (Figure 2B). Surprisingly, CD73 activity subsequently decreased with longer duration of isoflurane treatment (Figure 2A). Furthermore, transient increase in CD73 activity in EA.hy9262 cells did not require the induction of new CD73 synthesis as CD73 mRNA (Figure 2C) and protein (Figure 2D) did not increase with isoflurane treatment in these cells. Therefore, our studies show that isoflurane transiently increases CD73 activity without inducing new CD73 synthesis in cultured endothelial cells.

### Isoflurane Treatment Release Endothelial Cells Microparticles Containing CD73

**Figure 3:**
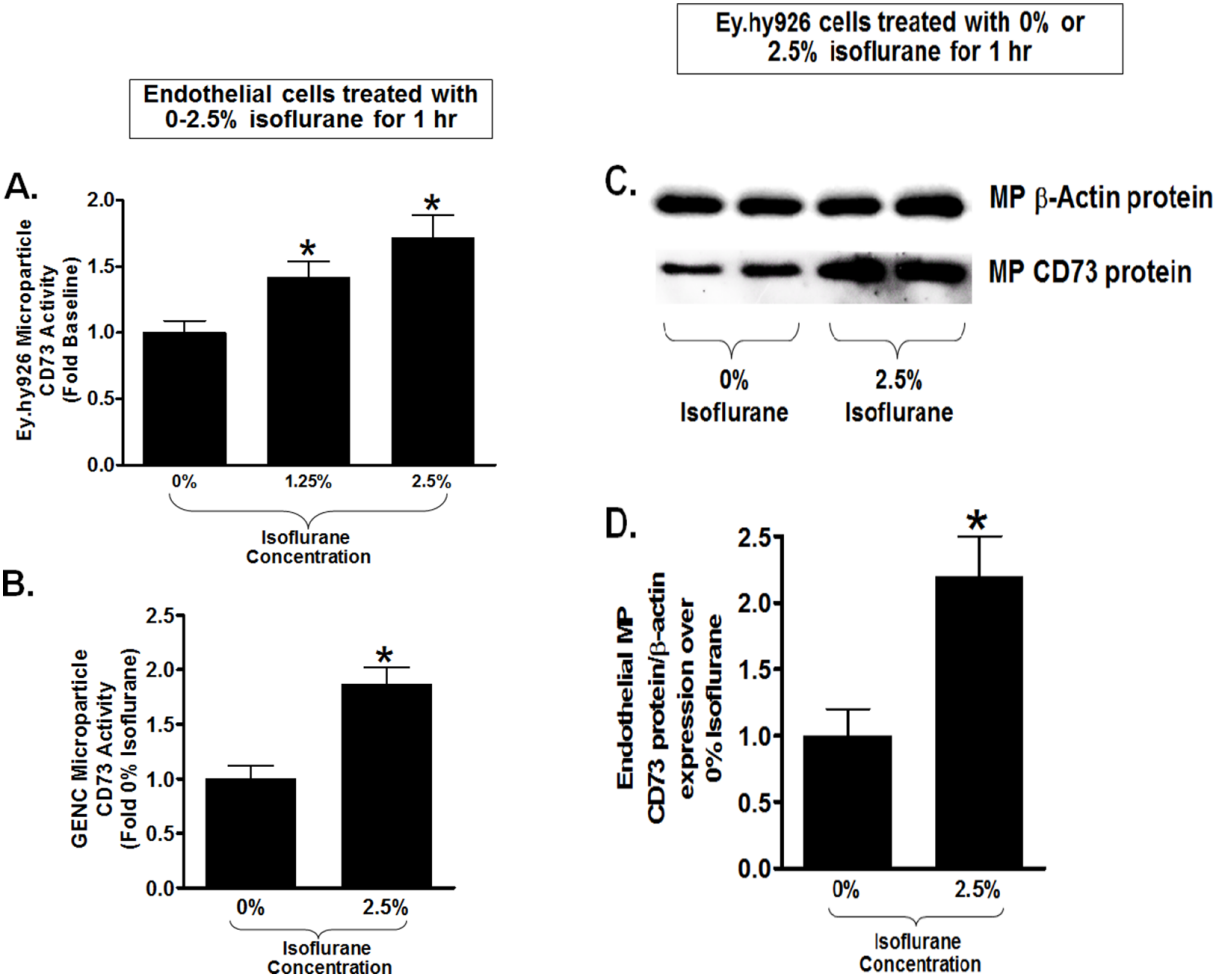
Isoflurane releases CD73 containing microparticles in cultured endothelial cells. A. Human umbilical vein endothelial (EA.hy926) cells were treated with 0–2.5% isoflurane for 1 hr and endothelial cell culture media microparticles (MP) were isolated and assayed for CD73 activity. Isoflurane caused a significant increase in human endothelial cell microparticle CD73 activity (N = 5–8). B. Microparticles isolated from mouse glomerular endothelial cells (GENC) treated with 2.5% isoflurane for 1 hr also had higher CD73 activity compared to carrier gas-treated cells (N = 4–6). C and D. Representative CD73 immunoblotting images (C) and band intensity quantifications (D) from microparticles isolated from EA.hy926 cells. Beta-actin protein expression was also quantified to normalize lane loading. Isoflurane treatment (2.5% for 1 hr) significantly increased CD73 protein expression in EA.hy926 cell microparticles compared to carrier gas-treated cells. *P<0.05 vs. carrier gas group. Error bars represent 1 SEM.

**Figure 4:**
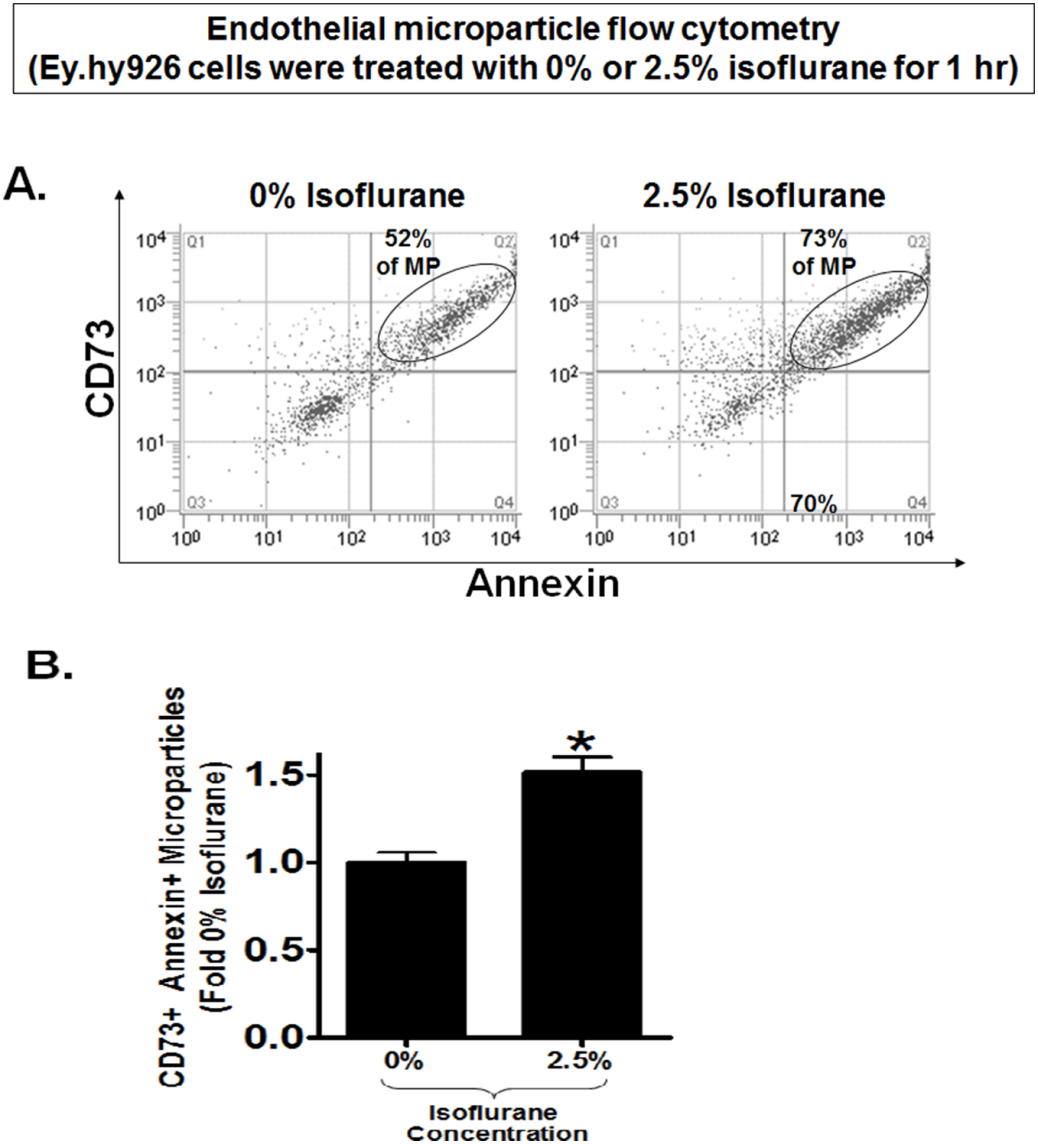
Flow cytometric analyses of endothelial cell culture media microparticles. A. Representative flow cytometric analyses of microparticles isolated from EA.hy926 endothelial cell culture media. EA.hy926 cells were treated with 2.5% isoflurane or with carrier gas for 1 hr and isolated microparticles were incubated with CD73 antibody and Annexin V. B. EA.hy926 cells treated with 2.5% isoflurane for 1 hr had significantly higher CD73+ Annexin V+ microparticles compared to microparticles isolated from carrier gas-treated endothelial cells (N = 5). *P<0.05 vs. carrier gas group. Error bars represent 1 SEM.

Since isoflurane transiently increases cell plasma membrane CD73 activity without inducing new CD73 synthesis in cultured endothelial cells, we tested whether isoflurane induces the release of plasma membrane microparticles containing CD73. Indeed, microparticles isolated from cell culture media of EA.hy9262 cells treated with 1.25 or 2.5% isoflurane for 1 hr had significantly increased CD73 activity compared to microparticles isolated from carrier gas-treated cells (Figure 3A). Isoflurane (2.5%) treatment for 1 hr also increased CD73 activity in microparticles isolated from GENC media (Figure 3B). Finally, microparticles from endothelial cells treated with 2.5% isoflurane had significantly increased CD73 protein expression compared to microparticle isolated from carrier gas-treated endothelial cells (Figure 3C and 3D). Flow cytometric analyses confirmed that endothelial cells treated with 2.5% isoflurane for 1 hr had significantly (∼50%) higher CD73+ Annexin V+ microparticles compared to microparticles isolated from carrier gas-treated endothelial cells (Figure 4).

### Isoflurane Increases CD73 Containing Endothelial-derived Microparticles in Mouse Plasma

**Figure 5:**
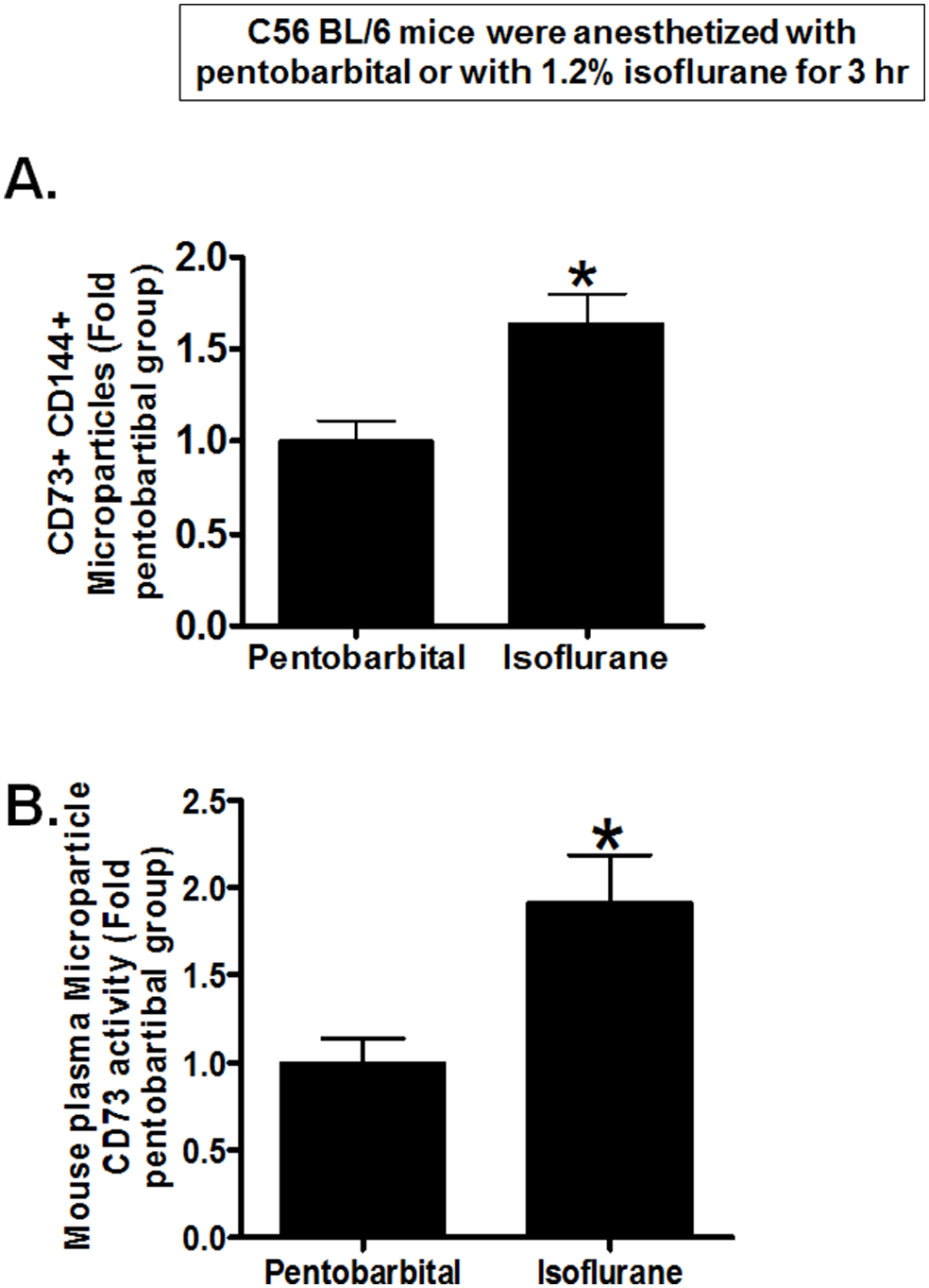
Isoflurane increases CD73 containing endothelial cell-derived microparticles in mouse plasma. Mouse plasma microparticles (MP) were isolated after 1.2% isoflurane or equi-anesthetic dose of pentobarbital anesthesia for 3 hr. A. Mice anesthetized with isoflurane had significantly increased CD144 (endothelial cell marker)+ CD73+ plasma microparticles compared to mice anesthetized with pentobarbital (N = 4). B. Plasma microparticles isolated from mice anesthetized with isoflurane had significantly higher CD73 activity when compared to CD73 activity in microparticles isolated from pentobarbital-anesthetized mice (N = 5). *P<0.05 vs. carrier gas group. Error bars represent 1 SEM.

We next determined whether isoflurane anesthesia increases endothelial cell-derived CD73 containing microparticles in plasma of mice anesthetized with isoflurane. Figure 5A shows that plasma of mice anesthetized with 1.2% isoflurane for 3 hr had significantly increased CD144 (an endothelial cell marker)+ CD73+ microparticles compared to microparticles isolated from mice anesthetized with equi-anesthetic dose of pentobarbital. Furthermore, plasma microparticles isolated from mice anesthetized with isoflurane had significantly higher CD73 activity when compared to CD73 activity in plasma microparticles isolated from pentobarbital-anesthetized mice (Figure 5B).

### Isoflurane Reduces Endothelial Cell Apoptosis and Inflammation via Induction of Endothelial Microparticle CD73

**Figure 6:**
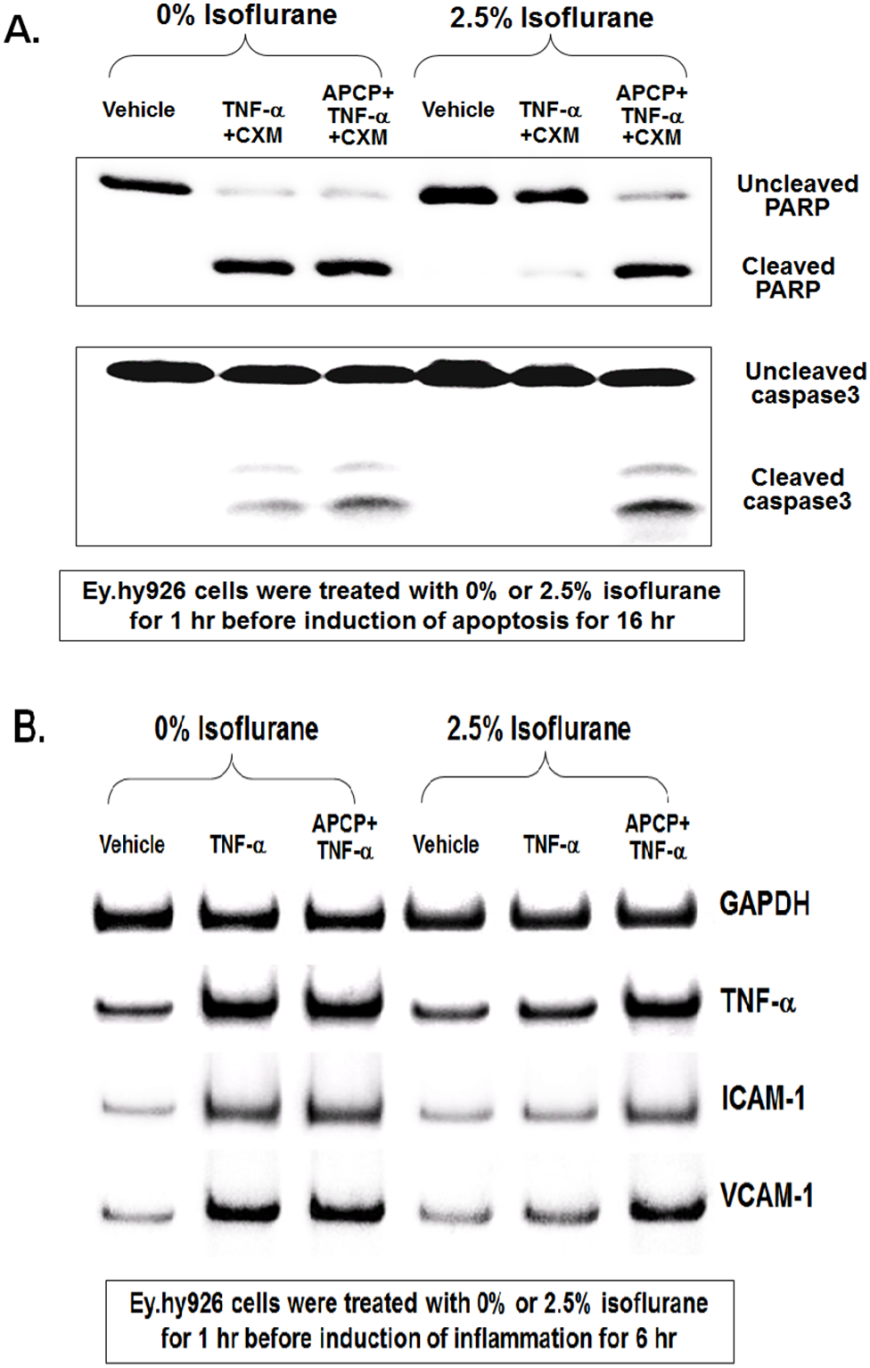
Isoflurane reduces endothelial cell apoptosis and inflammation via CD73. A. Representative immunoblot of poly(adenosine diphosphate-ribose) polymerase (PARP) and caspase-3 fragmentation (N = 4) as indices of EA.hy926 endothelial cell apoptosis induced by TNF-α (20 ng/ml) and cycloheximide (CHX; 10 µg/ml) treatment for 16 hr. EA.hy926 cells treated with TNF-α and cycloheximide for 16 hr had robust PARP and caspase-3 fragmentation. In contrast, EA.hy926 cells treated with 2.5% isoflurane for 1 hr before the induction of apoptosis isoflurane showed reduced apoptotic death indicated by decreased PARP and caspase-3 fragmentation. Supporting a critical role of CD73 in isoflurane-mediated protection against EA.hy926 cell apoptosis, cells pretreated with a selective CD73 inhibitor APCP (100 µM) were not protected against endothelial apoptosis with isoflurane pretreatment. B. Representative gel images of pro-inflammatory mRNA expression (VCAM-1, ICAM-2 and TNF-a) in EA.hy926 endothelial cells treated with TNF-α (20 ng/ml) for 6 hr. EA.hy926 cells treated with TNF-α had increased mRNA encoding markers of inflammation. In contrast, EA.hy926 cells treated with 2.5% isoflurane for 1 hr before TNF-α treatment had reduced inflammatory mRNA expression. Again, supporting a critical role of CD73 in isoflurane-mediated protection against EA.hy926 cell inflammation, cells pretreated with a selective CD73 inhibitor APCP were not protected against endothelial inflammation with isoflurane pretreatment.

We then tested whether CD73 is critical for isoflurane-mediated protection against endothelial apoptosis and inflammation. EA.hy9262 cells pretreated with carrier gas underwent apoptosis with robust PARP and caspase-3 fragmentation (Figure 6A) after TNF-α and cycloheximide treatment for 16 hr. In contrast, EA.hy9262 cells pretreated with 2.5% isoflurane for 1 hr had reduced apoptotic death indicated by decreased PARP and caspase-3 fragmentation. Supporting a critical role of CD73 activation in isoflurane-mediated protection against endothelial cell apoptosis, cells pretreated with a selective CD73 inhibitor APCP (100 µM) were not protected against endothelial apoptosis with isoflurane treatment (Figure 6A). Endothelial cells treated with TNF-a for 6 hr showed induction of several pro-inflammatory mRNAs including TNF-α, ICAM-1 and VCAM-1. Isoflurane pretreatment reduced the upregulation of these pro-inflammatory mRNAs. Again supporting a critical role of endothelial CD73 in isoflurane-mediated reduction in inflammation, endothelial cells pretreated with a selective CD73 inhibitor APCP (100 µM) were not protected against endothelial inflammation with isoflurane treatment (Figure 6B).

### Isoflurane-mediated Activation of Rho Kinase Mediates the Release of Endothelial Microparticles Containing CD73

**Figure 7:**
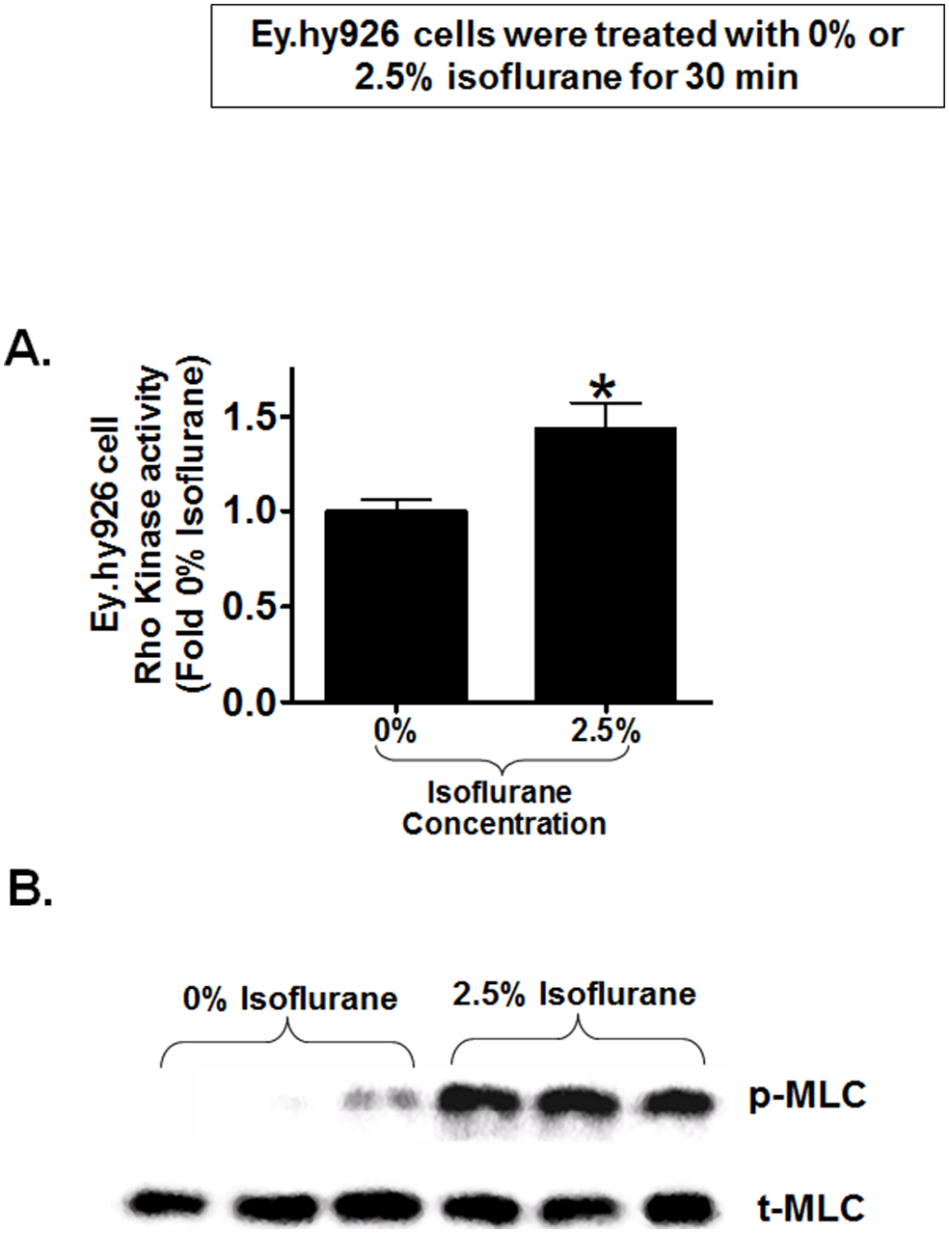
Isoflurane stimulates Rho kinase activity in human endothelial cells. A. Rho kinase activity in human endothelial (EA.hy9262) cells was measured by detecting myosin phosphatase target protein-1 phosphorylation after treatment with 2.5% isoflurane or with carrier gas for 30 min. Isoflurane significantly increased Rho kinase activity in EA.hy9262 cells compared to carrier gas-treated cells (N = 5). *P<0.05 vs. carrier gas group. Error bars represent 1 SEM. B. EA.hy9262 endothelial Rho kinase activity was also assessed by detecting myosin light chain (MLC) phosphorylation in EA.hy9262 cells with immunoblotting. MLC phosphorylation increased in EA.hy9262 cells treated with 2.5% isoflurane for 30 min compared to carrier gas-treated cells. Total MLC immunoreactivity did not change with isoflurane treatment. Representative of 2 experiments performed in triplicate.

**Figure 8:**
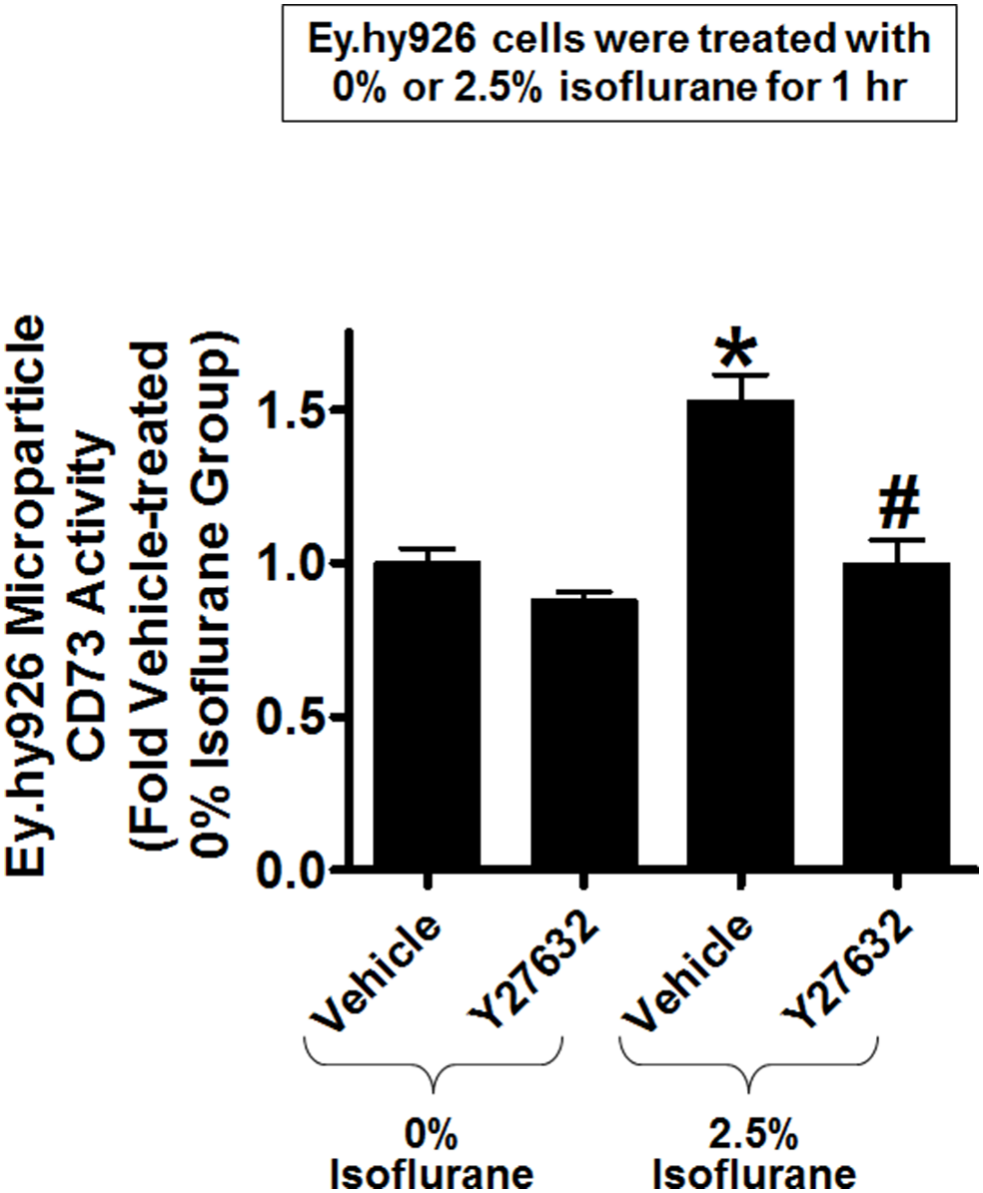
Isoflurane-mediated activation of Rho kinase mediates the release of CD73 containing endothelial microparticles. Human endothelial (EA.hy9262) cells were treated with 2.5% isoflurane or with carrier gas for 1 hr. Some cells were pretreated with a selective Rho kinase inhibitor Y27632 for 30 min. before isoflurane or carrier gas treatment. Y27632 prevented the isoflurane-mediated increase in microparticle CD73 activity in EA.hy9262 cells (N = 4). *P<0.05 vs. carrier gas group treated with vehicle. #P<0.05 vs. isoflurane group treated with vehicle. Error bars represent 1 SEM.

Since previous studies showed that Rho kinase activation increases endothelial microparticle generation [28]–[30], we tested the hypothesis that isoflurane activates endothelial Rho kinase to release endothelial microparticles containing CD73. Figure 7A shows a significant increase in Rho kinase activity (detected by MYPT-1 phosphorylation) in EA.hy9262 cells treated with 2.5% isoflurane for 30 min. compared to carrier gas. Furthermore, Figure 7B shows increased p-MLC immunoreactivity in EA.hy9262 cells treated with 2.5% isoflurane for 30 min. Total MLC immunoreactivity did not change with isoflurane treatment. In addition, we were able to block isoflurane-mediated MLC phosphorylation with a selective inhibitor of Rho kinase (Y27632, data not shown). Finally, Figure 8 shows that a selective Rho kinase inhibitor Y27632 prevented isoflurane-mediated increases in microparticle CD73 activity in EA.hy9262 cells supporting a critical role for Rho kinase in isoflurane-mediated release of CD73 containing endothelial microparticles.

## Discussion

Overactive inflammatory response is a detrimental complication of surgery and perioperative surgical infections. Anti-inflammatory and cytoprotective effects of volatile inhalational anesthetics are well recognized [31], [32]. However, the mechanisms mediating the protective effects of volatile anesthetics have not been fully elucidated. Our findings suggest that volatile anesthetics, at least in part, attenuate endothelial inflammation and apoptosis by releasing CD73 containing endothelial microparticles. We found that clinically relevant concentrations of isoflurane (1.25–2.5%) increased adenosine generation and rapidly increased endothelial CD73 activity in cultured human umbilical vein endothelial and mouse glomerular endothelial cells. We were surprised to discover that isoflurane-mediated induction of CD73 activity was transient and occurred without any changes in CD73 expression. We determined that isoflurane-mediated induction of CD73 activity was due to the release of preformed CD73 contained in endothelial plasma membrane microparticles. CD73 activity was critical for isoflurane-mediated protection against endothelial apoptosis and inflammation. Finally, the mechanisms of isoflurane-mediated release of CD73 containing microparticles are mediated by activation of endothelial Rho kinase.

In addition to its analgesic and anesthetic properties, volatile anesthetics have non-anesthetic effects in virtually every cell type. Importantly, volatile anesthetics protect against cell death and inflammation in several key organs including the heart, brain, kidney and intestine. For example, several clinically utilized volatile anesthetics including isoflurane precondition the heart against ischemia and reperfusion injury [11]. We have previously demonstrated that volatile anesthetics including isoflurane display anti-necrotic and anti-inflammatory effects in renal proximal tubule cells *in vivo* and *in vitro* [5], [12], [13]. Harnessing these non-anesthetic properties of volatile anesthetics may have important clinical implications for critically ill patients anesthetized in the OR and sedated in the ICU.

Previous studies have shown that isoflurane anesthesia in mice and rats attenuates lipopolysaccharide induced endothelial inflammation [16], [17]. Furthermore, isoflurane inhalation after LPS injection in rats *in vivo* attenuates systemic cytokine (IL-1β and IL-6) upregulation and well as lung injury [33], [34]. We previously showed that volatile anesthetic isoflurane protect against human endothelial apoptosis and inflammation [13], [35]. Consistent with our findings, others have also shown that isoflurane treatment attenuated lipopolysaccharide or TNF-α induced endothelial cell inflammation and death *in vitro* [14], [36]. Although the robust anti-inflammatory and protective effects of isoflurane have been demonstrated *in vivo* and *in vitro*, the detained cytoprotective mechanisms remained elusive.

We recently showed that isoflurane protected against renal tubular necrosis, apoptosis and inflammation by direct induction of CD73 enzyme and activity leading to enhanced adenosine generation [18]. CD73 is a well-known anti-inflammatory and anti-ischemic enzyme. Mice deficient in CD73 have increased tissue and vascular inflammation and have a higher mortality rate after ischemia and reperfusion injury and sepsis [37], [38] [39]. Moreover, enhanced CD73 activity protects against intestinal, cardiac and renal ischemia reperfusion injury [37], [38], [40]. Cell surface CD73 catalyzes the hydrolysis of AMP to adenosine and is a critical step in extracellular adenosine generation [41]. Extracellular adenosine regulates diverse and important physiological effects including cardiac inotropy and chronotropy, vascular tone and kidney glomerular filtration rate. Furthermore, adenosine protects against tissue injury and inflammation after ischemia and reperfusion or sepsis. Adenosine acts via activation of 4 G-protein coupled purinergic receptors [A_1_, A_2a_, A_2b_ and A_3_ adenosine receptors] [41], [42]. In particular, activation of A_1_, A_2a_ or A_2b_ARs protects against ischemia reperfusion injury in the kidney, heart, liver and brain [43], [44]. Unlike findings in renal proximal tubular cells where CD73 synthesis was increased after isoflurane treatment, isoflurane increases adenosine generation in endothelial cells by releasing preformed CD73 contained in endothelial plasma membrane microparticles without synthesizing new CD73 enzyme [18]. The exact subtype(s) of adenosine receptor(s) involved in endothelial protection by isoflurane-mediated adenosine generation remains to be elucidated.

**Figure 9:**
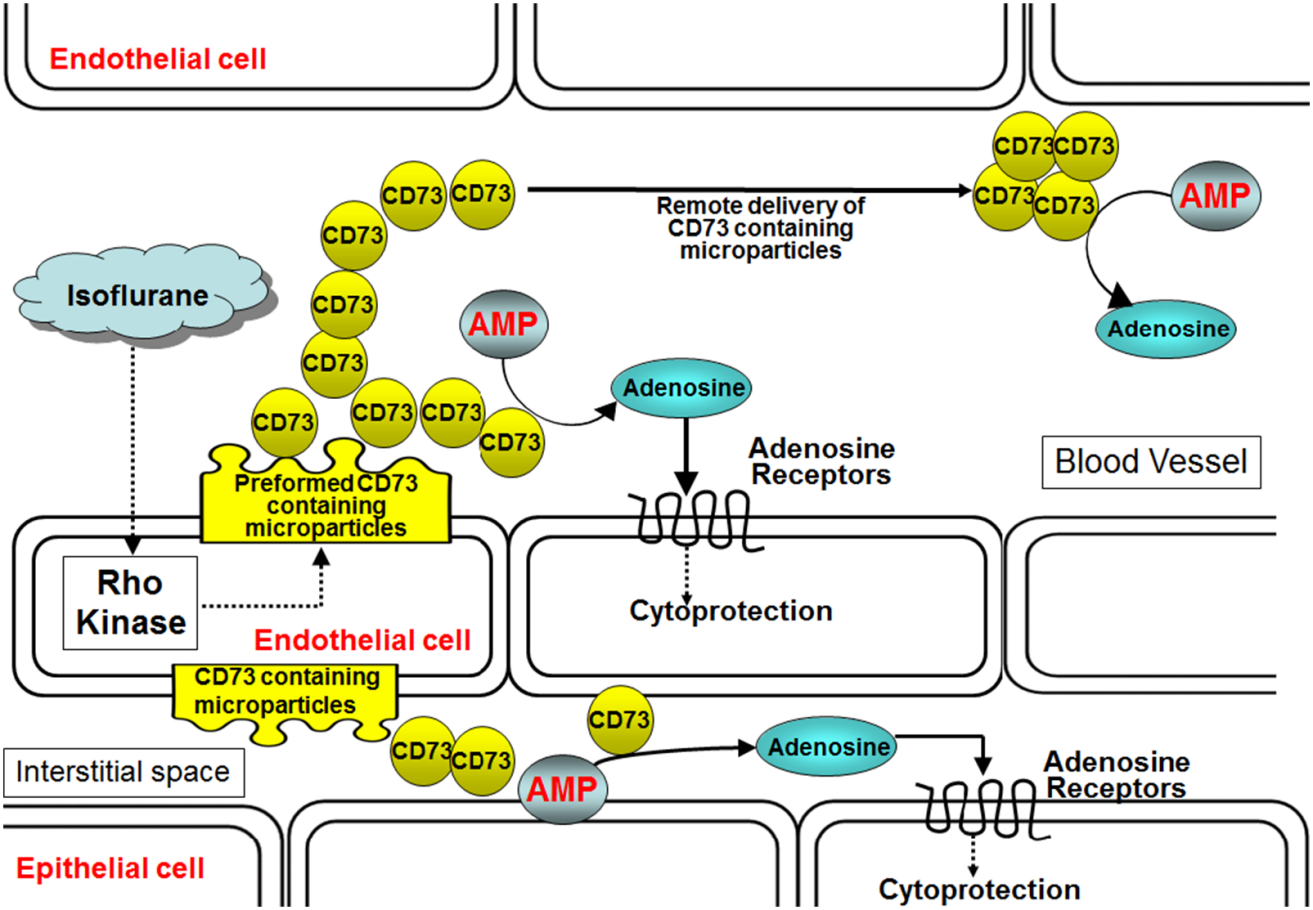
Proposed mechanisms of isoflurane-mediated endothelial CD73 generation. We hypothesize that isoflurane via Rho kinase activation releases endothelial microparticles containing CD73. Increased endothelial microparticle CD73 converts AMP to adenosine which produces cytoprotective effects on neighboring endothelial cells or renal epithelial cells via activation of adenosine receptors. Furthermore, we hypothesize that remote delivery of CD73 containing microparticles may provide systemic anti-inflammatory effects of isoflurane anesthesia. Abbreviations: AMP = adenosine monophosphate, CD73 = ecto-5′-nucleotidase.

In this study, we demonstrate that isoflurane rapidly released endothelial microparticles containing preformed CD73 in cultured endothelial cells as well as in plasma of mice. Indeed, CD73 was directly responsible for isoflurane-mediated endothelial cell protection. Plasma membrane microparticles are phospholipid microvesicles of submicron (0.1 to 1.0 µm) fragments that originate from plasma membrane blebbing and are subsequently shed [4], [45]. Microparticles play an important role in the transfer of materials between cells. Furthermore, they are critical in transferring signaling information to cells close by or far away. Plasma microparticles are elevated in several pathological conditions including vascular thrombosis, hyperlipidemia, diabetes, chronic renal dysfunction and cancer [4], [28], [46]. Here we show that endothelial microparticles could also have a cytoprotective and beneficial role. We propose that isoflurane treatment propagates the systemic release of endothelial microparticles containing active CD73 that function as cytoprotective messengers by generating adenosine. Isoflurane-mediated endothelial microparticle generation may prevent damage and favor vascular repair by preventing endothelial apoptosis and inflammation. Furthermore, microparticle-mediated delivery of CD73 allows adenosine formation in different cell types (e.g., epithelial cell). Finally, microparticle generation allows remote delivery of CD73 away from the originating endothelial cell. Our findings imply that isoflurane-mediated generation of CD73 containing microparticles in one organ (e.g., lung) may travel to distant locations (e.g., kidney, liver) to produce multi-organ anti-inflammatory effects (Figure 9).

We also demonstrate an important role for Rho kinase activation in isoflurane-mediated CD73 containing endothelial microparticle release. We show in this study that isoflurane-mediated endothelial microparticle release and induction of CD73 activity were significantly attenuated by a selective Rho kinase inhibitor. In many cell types including endothelial cells, Rho kinase regulates cytoskeleton architecture, migration and growth [47], [48]. Previous studies suggest that volatile anesthetics including isoflurane activate Rho kinase and promote Rho A function. In primary neuronal cultures as well as rat glioma C6 cell line, Rho A was activated after exposure to ∼1 MAC isoflurane [49], [50]. Evidence for Rho kinase-mediated endothelial microparticle formation and release also provided by Burger *et al* [28]. They showed that angiotensin II-mediated microparticle formation is mediated by Rho kinase pathways targeted to lipid rafts. In addition, thrombin induces endothelial microparticle generation via Rho kinase activation [29]. Finally, endothelial microparticles released with TNF-α treatment in human coronary artery endothelial cells are suppressed by a specific Rho kinase inhibitor (Y-27632) [30].

In summary, we demonstrate that a commonly utilized volatile anesthetic isoflurane rapidly increases endothelial cell adenosine generation via releasing microparticles containing preformed CD73. Release of CD73 and subsequent adenosine generation may result in cellular protection in neighboring and remote endothelial and epithelial cells via activation of adenosine receptors. Our current findings in endothelial cells differ considerably from findings in renal tubular epithelial cells as increased adenosine generation occurred without the induction of new CD73 synthesis. Taken together, our current study provides additional mechanistic insight into the mechanism of isoflurane-mediated endothelial cell protection.
